# Right Ventricular Thrombus: A Rare Complication of Ovarian Hyperstimulation Syndrome

**Published:** 2012-12-15

**Authors:** Mahmood Zamirian, Ali Reza Moaref, Seyed Hosein Alavi, Khalil Zarrabi

**Affiliations:** 1 Cardiovascular Research Center, Shiraz University of Medical Sciences, Shiraz, IR Iran

**Keywords:** Ovarian Hyperstimulation, Thrombosis

## Abstract

A 22 years old lady was admitted because of progressive dyspnea, severe abdominal protrusion and lower extremity edema. She had undergone ovarian hyperstimulation for primary infertility by Clomiphen and Human chorionic gonadotropin for 3 months. Abdominopelvic ultrasonography revealed bilateral enlarged multi cystic ovaries and massive ascites. Transesophageal echocardiography revealed a large thrombus in right ventrice apex. Spiral chest CT scan showed normal pulmonary vasculature with no evidence of pulmonary thromboembolism. Heparin was started and repeat echocardiographic study showed gradual disappearance of right ventricular thrombus. Human chorionic gonadotropin is the most important substance which leads to capillary leakage and fluid accumulation in third space. Fluid shift and hypovolemia may cause hypotension, hemoconcentration and formation of vascular thrombus.

## 1. Introduction

Ovarian hyperstimulation syndrome is a well-known entity in assisted reproductive technique. It has frequent complications. Venus and arterial thrombosis are the most dangerous complications. A case with ovarian hyperstimulation syndrome with right ventricular thrombus is presented.

## 2. Case Report

A 22 years old lady was admitted because of progressive dyspnea, severe abdominal protrusion and lower extremity edema. She had experienced gradual abdominal pain and dyspnea at rest since one week before admission. Ovarian hyperstimulation had been performed for primary infertility by Clomiphen and Human chorionic gonadotropin (HCG) for 3 months. Her gynecologist had stopped these medications, but as her symptoms aggravated, she was admitted to ICU. She denied any previous cardiovascular disease. Her physical examination exhibited BP 90/60, PR:108, RR 24/min normal jugular venous pressure, reduced breathing sounds in both lung bases , normal cardiac sounds, marked abdominal protrusion, tender abdomen and 2 + lower extremity edema .

**Figure 1.**
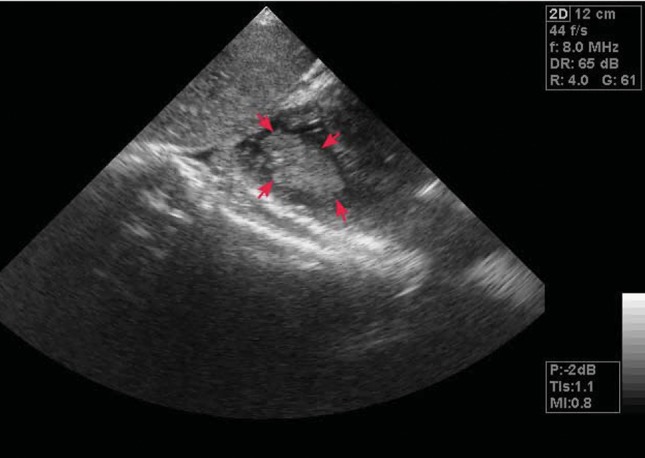
A Large Thrombus in Right Ventricular Apex

Paraclinical work up revealed: Hb: 12.8 g/dl; Hct: 48; WBC: 24000/mm3 BUN, Creatinine and liver function test were normal and ß-HCG was 25400pg/mm3 Chest X-Ray showed bilateral plural effusion. Abdominopelvic ultrasonography demonstrated bilateral enlarged multi-cystic ovaries and massive ascites. Because of patient’s dyspnea and poor echo window, she underwent transesophageal echocardiography that exhibited a large thrombus in right ventricular apex (Figure1). Color Doppler study of both lower extremities and pelvic veins failed to show any thrombus in venous system, and spiral chest Computed Tomography Scan showed normal pulmonary vasculature and no evidence of pulmonary thromboembolism. Dose-adjusted heparin was started with concurrent supportive care. Follow up echocardiographic study revealed gradual disappearance of right ventricular thrombus. She was finally discharged after 10 days in good general condition.

## 3. Discussion

Assisted reproductive technique and other infertility treatment can frequently lead to ovarian hyperstimulation syndrome. Intravascular shift of fluid into peritoneal, pleural and pericardial space is known to be due to production and release of vascular endothelial growth factor (EDGF) by stimulated ovaries. The vaso-active substances such as interleukins and tumor necrosis factor alpha and endothelin-1 are other known culprits(1). HCG is the most important substance which leads to capillary leakage and fluid accumulation in the third space. Ovarian torsion, rupture of ovarian cysts and peritonitis are other abdominal complications. Fluid shift and hypovolemia may culminate in hypotension, hemoconcentration and vascular thrombus formation(2). High estrogen level may increase platelet count; fibrinogen level and Von Wilebrand factor (3).

Hemoconcentration, hypovolemia and high estrogen level with or without inherited thrombophilia including anti-thrombin III deficiency, protein C and S deficiency, anti-phospholipid antibody syndrome and factor V laden mutation, are thought to be the main causes of thrombus formation. Thromboembolic event is a very dangerous manifestation of hyperstimulation syndrome with estimated incidents of 0.4% (4). Another study on subjects aged over 39 years with hyper-homocysteinemia showed strong association with occurrence of thrombotic complications during in-vitro fertilization(4). Both arterial and venous system can be involved in thrombotic occlusion. Intracranial thrombosis and jugular, subclavian and lower extremity venous system are usual sites of involvement. Pulmonary embolism has rarely been reported(5). Acute myocardial infarction due to thrombosis in coronary artery is also a rare complication(6). Obstetrician and internist should be aware of these thrombotic complications of ovarian hyperstimulation syndrome which may occur during the course of treatment or even weeks after resolution of symptoms(7). To the best of our knowledge, there is no reported case of right ventricular thrombosis as a complication of ovarian hyperstimulation syndrome.
